# Research on Energy Management in Forward Extrusion Processes Based on Experiment and Finite Element Method Application

**DOI:** 10.3390/ma18112616

**Published:** 2025-06-03

**Authors:** Tomasz Miłek, Olga Orynycz, Jonas Matijošius, Karol Tucki, Ewa Kulesza, Edward Kozłowski, Andrzej Wasiak

**Affiliations:** 1 Faculty of Mechatronics and Mechanical Engineering, Kielce University of Technology, Al. Tysiąclecia Państwa Polskiego 7, 25-314 Kielce, Poland; tmatm@tu.kielce.pl; 2 Department of Production Management, Faculty of Engineering Management, Bialystok University of Technology, Wiejska Street 45A, 15-351 Bialystok, Poland; 3 Mechanical Science Institute, Vilnius Gediminas Technical University, Plytinės Str. 25, LT-10105 Vilnius, Lithuania; jonas.matijosius@vilniustech.lt; 4 Department of Production Engineering, Institute of Mechanical Engineering, Warsaw University of Life Sciences, Nowoursynowska Street 164, 02-787 Warsaw, Poland; 5 Department of Mechanics and Applied Computer Science, Faculty of Mechanical Engineering, Bialystok University of Technology, Wiejska Street 45A, 15-351 Bialystok, Poland; ewa.kulesza@pb.edu.pl; 6 Faculty of Management, Lublin University of Technology, Nadbystrzycka 38, 20-618 Lublin, Poland; e.kozlovski@pollub.pl; 7 Advisors Panel Production Engineering, Alternative Energy Sources Bialystok, 15-351 Bialystok, Poland; andrzej.wasiak@gmail.com

**Keywords:** energy efficiency in manufacturing, forward extrusion process optimization, finite element method in metal forming, decision-making in industrial processes, predictive modeling for process optimization

## Abstract

This paper advances the forward extrusion process by integrating sustainable methodologies and optimizing energy efficiency. This research investigates the impact of die geometry and elongation coefficients on energy usage and process efficiency, employing finite element method (FEM) simulations alongside empirical analysis. Artificial neural networks and experimental data were utilized to predict process energy. The experimental study utilized flat, conical, and arc-shaped dies to extrude lead profiles exhibiting different elongation coefficients. The study analyzed the dynamics of material flow, energy requirements, and maximum forces. Patterns of deformation, distribution of tension, and losses of energy were discerned, with finite element models enhancing understanding of these phenomena. The mathematical framework forecasting the peak extrusion force in relation to elongation parameters was substantiated via residual diagnostics and regression analysis. The findings indicate that conical and arc dies can conserve up to 15% of the energy in comparison to flat dies, thereby improving material flow and reducing deformation forces. This comprehensive strategy provides practical solutions to reduce energy consumption and improve metal forming processes, thereby enhancing industrial efficiency and sustainability. The results not only benefit industry but also align with environmental objectives, thereby increasing the efficiency and sustainability of extrusion operations.

## 1. Introduction

Emphasizing the need for creative energy-saving solutions, the metal forming sector—especially extrusion operations—remains among the most energy-intensive sectors [1,2]. Moreover, the choice of die parameters—like geometry and elongation coefficient—strongly affects energy usage during extrusion operations. Planning extended production series depends very much on these factors; slight inefficiencies can cause significant cumulative energy demand. Recent research has underlined the important parts material flow behavior and tool shape play in energy-intensive processes like forward extrusion. For example, the finite element method (FEM) has been shown to be a useful tool for simulating material stress and energy requirements, thereby enabling the design of more efficient equipment and processes [3]. Research on hybrid systems integrating additive manufacturing (AM) with traditional techniques such as sheet forming has also show great promise for lowering energy consumption and raising product quality [4,5].

Recent studies emphasize the importance of optimizing die design and process parameters to improve energy efficiency in forward extrusion. For instance, Barisic and Cukor [6] modeled energy consumption in backward extrusion and highlighted the sensitivity of results to friction assumptions. Bakhshi-Jooybari [7] demonstrated the challenges of accurate friction modeling in hot and cold extrusion of various materials. Moreover, hybrid approaches integrating numerical modeling with experimental calibration have proven effective in enhancing model accuracy [3]. The use of artificial intelligence, such as neural networks, further allows for predictive analysis of energy demand, especially when experimental data is scarce or costly to obtain [8]. These developments underscore the need to integrate physical modeling, machine learning, and real-time sensing in extrusion optimization.

Adoption of modern monitoring systems has changed manufacturing sector energy management. Real-time energy consumption data from IoT sensors and devices helps firms to find inefficiencies and flexibly change operations. For example, sensors fitted in important systems can identify anomalies in energy consumption, therefore enabling quick action and minimizing waste [8,9]. By allowing predictive analytics, machine learning techniques and artificial intelligence (AI) improve energy management even further. These systems are capable of forecasting energy consumption, optimizing machine scheduling, and projecting maintenance requirements through the analysis of historical data. This proactive strategy enhances overall efficiency [10,11,12], reduces unplanned downtime, and facilitates more sensible resource allocation. Increasingly linked with IoT systems, AI-based technologies create intelligent ecosystems that continuously monitor and maximize energy use in real-time. Modern manufacturing methods are characterized mostly by their capacity to strike a compromise between sustainability and efficiency. The mix of AM with traditional production processes has further raised the prospect of energy savings. Combining AM with sheet forming and other hybrid processes has shown how well materials are exploited and how low thermal residual stresses are attained. These techniques especially help to generate intricate geometries with improved dimensional accuracy, hence lowering the demand for post-processing and further energy inputs [13,14]. Since they provide exact knowledge of material flow, deformation forces, and energy dynamics during the extrusion process in this field, FEM simulations have become indispensable [6,15]. Innovations in material science are continually driving sustainability in production ever. One example of how circular economy ideas could be used in industrial operations is the use of recycled materials—such as abrasives in polishing operations. Recycled abrasives have lower energy usage and costs, while meeting or exceeding the performance of standard materials [16,17]. Hybrid composite materials are gaining popularity due to their high energy efficiency and effectiveness. Steel-reinforced aluminum foams have good strength and efficient energy absorption capabilities. These materials are suitable for applications necessitating robust structures and energy efficiency, such as in automobiles and aircraft [18]. The optimization of volumetric energy density has emerged as a primary area of research in additive manufacturing. This value directly affects the mechanical properties of produced components and energy usage. Careful regulation of energy density has been shown in studies to attain a perfect balance between efficiency and part quality, therefore supporting more sustainable manufacturing methods [13,19]. Advanced algorithms such as the Multimodal Attention Fusion Network (MAFN) help manufacturers to combine intricate data sets for real-time optimization, therefore lowering energy consumption and enhancing output of manufacturing [20]. Using soft metals such as lead requires understanding of plastic flow to optimize operations. Lead deforms polymers even under relatively mild strains due to its face-centered cubic crystalline structure. Multiple theories elucidate the behavior of lead during plastic deformation: the Arrhenius equation illustrates the correlation between deformation rate and temperature, while the Bailey–Norton law pertains to long-term creep behavior. These models provide useful information about the energy dynamics of metal forming processes [21]. Plastic deformation processes include elastic deformation, heat dissipation, and permanent structural changes in terms of energy. The majority of energy input for lead is converted into heat and plastic deformation, with relatively little elastic contribution. While Taylor’s law stresses the importance of dislocation density in determining the stress required for additional deformation [22], advanced models such the Huber–Mises–Hencky energy criterion offer more insight on how important critical strain energy levels initiate deformation [23,24].

One of the basic metal processing processes is extrusion [25,26]. In the extrusion process, heated metal (charge) is introduced into the die [27,28]. It is then subjected to high pressing forces using a die. As a result, the metal undergoes plastic deformation, adapting to the shape of the matrix [29,30]. The extrusion process is the subject of intense scientific research. In [31], pure zinc rods were subjected to forward extrusion (FE) and composite extrusion (CE) at 120 °C to investigate changes in microstructure and mechanical properties. The study found that grain growth and texture weakening lead to loss of strength, while the increase in plasticity after extrusion was attributed to the activation of more slip systems. The influence of initial deformations on the microstructure and mechanical properties of the AZ31 magnesium alloy was studied in [32]. The initial deformation process was added before alternating forward extrusion forming. It is noticed that, as the initial strain increases, the elongation at break tends to decrease and the tensile strength tends to increase [33]. Moreover, it has been proven that the strengthening of the initial deformation process not only makes the basal grain plane in the billet randomly distributed and weakens the basal plane texture, but also stores a large amount of deformation and displacement energy inside the billet, which accelerates the subsequent continuous dynamic recrystallization (CDRX) process) consisting of alternating extrusion [34].

Reference [35] analyzed the flow control at the exit of the extrusion die. For this purpose, a mathematical model of the process was developed. The model takes into account thermal effects and assumes that the properties of extruded material are strain rate and temperature dependent. Analytical sensitivities were developed from discrete finite element equations to calculate the necessary derivatives during optimization. Modeling friction forces at the interface between the die and the workpiece is the subject of research presented in [7]. This paper investigated friction modeling using an extrusion process and showed that the model performs very well for cold extrusion of dry aluminum, but does not adequately model hot extrusion of lubricated steel. The research results of the influence of friction coefficient, load and displacement investigated and analyzed in forward extrusion of AA6063 are presented in reference [36]. Friction coefficient values were found to vary significantly for large area reductions compared to minimal area reductions [37].

The aim of the research presented in this paper was to simulate stresses inside the pressed material during the plastic forming process by different die geometries. Using this methodology, the influence of the die geometry on the distribution of material flow lines in the cross-section was determined. The analysis was supplemented with experimental data from the actual process and numerical calculations in order to obtain the energy of a single process. In addition, models were created using artificial neural networks and experimental data to predict the energy of the least and most energy-consuming process.

The novelty of this research lies in the integrated use of experimental testing, finite element modeling, and artificial neural networks to evaluate and predict energy consumption in forward extrusion processes. A regression-based mathematical model was developed and statistically validated to quantify the relationship between elongation factor and extrusion force. This approach enables more precise energy forecasting and supports the design of more efficient extrusion tools, contributing to sustainable manufacturing practices.

## 2. Materials and Methods

Computer modeling of the forward extrusion process was carried out using the commercial QFORM-2D v.10 software based on FEM and intended for simulating metal forming processes in the field of volume shaping. It was assumed that the die is stationary and the punch moves at a constant speed *v* = 20 mm/min.

The diameter of the punch was 40 mm and the diameter of the container was 40.5 mm. The diameter of the samples was 39 mm and the height was 37 mm. The experimental material used in the tests was lead with a purity of 99.9325% Pb, which was confirmed on the basis of the certificate in accordance with BS EN 1024: 2004 [38]. The chemical composition was analyzed by Royston Lead Limited (Great Britain). Lead is a material that can be and is widely used for the physical modeling of various metal forming processes, with particular emphasis on forging and extrusion of the material [39]. It is very soft and its recrystallization temperature is often below environment temperature. This is influenced by the fact that the deformation of lead at environment temperature is already hot plastic working. This is important in the process of material extrusion, where there are high values of compressive stresses affecting excessive load and reducing the tool life [40,41]. Lead is not a typical material used in the construction of machine components, but model tests carried out using it allow the formulating of conclusions that are helpful in the deformation of other materials, because the structure of lead is similar to the structure of alloy steels [42].

**Figure 1:**
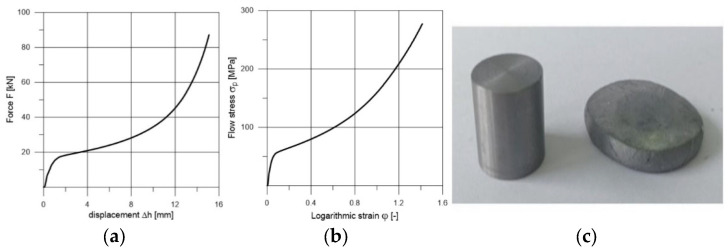
Example of test piece made from lead before and after upsetting: (**a**)—force vs. displacement; (**b**)—true flow stress vs. logarithmic strain; (**c**)—example of test piece before and after upsetting.

The test characteristics of lead presented in Figure 1a (force vs. displacement) and Figure 1b (true flow stress vs. logarithmic strain) for the purposes of simulation were developed in the process of upsetting samples on the ZD100 testing machine modernized by LABORTECH with a pressure of 1 MN, meeting the metrological requirements for class 1 in the field of measurement of forces and displacements, equipped with the Test&Motion software (version 5.5) to support experiments. An example of the test piece before and after upsetting is shown in Figure 1c.

**Figure 2:**
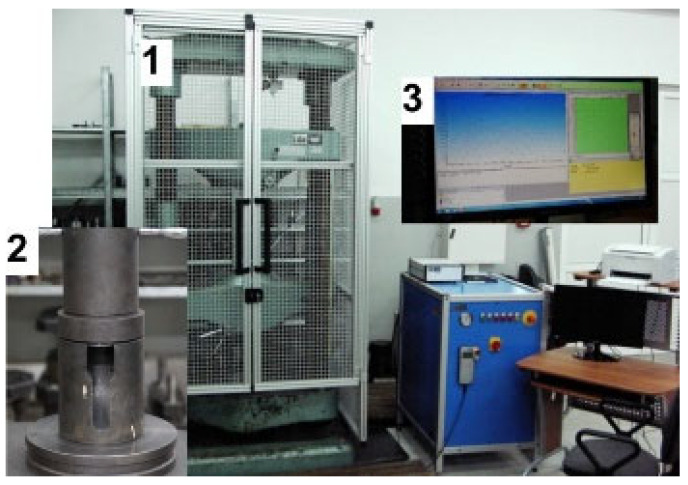
Stand for experimental tests of the forward extrusion process of solid profiles, where: 1—ZD100 testing machine (LABORTECH), 2—extrusion tooling, 3—Test&Motion software (LABORTECH).

The contact conditions between the material and the tools were determined by using an available lubricant—mineral oil from the QFORM-2D program database, recommended and intended for forming non-ferrous metals [43,44]. The assumed friction coefficient was 0.15 and the effective heat transfer coefficient was 7500 W/(m^2^ K) [45]. Experimental verification of the forward extrusion process was carried out on the stand shown in Figure 2.

The equipment included a ZD100 testing machine (VEB Werkzeugmaschinen-Kombinat Fritz Heckert, Leipzig, Germany) with laboratory tooling for forward extrusion of solid profiles with the possibility of changing dies. The experimental results regarding changes in pressure forces as a function of punch displacement were recorded using the Test&Motion software. In experimental investigations, before the forward extrusion process, the billets were lubricated using mineral oil with molybdenum disulfide.

Numerical integration using Newton–Cotes quadratures based on the Lagrange interpolation polynomial was used to determine the energy [46,47]. The work of the system was numerically determined as the surface area under the graph, based on the discrete dependences of the pressure force on the displacement of the punch. Then the basis was the relationship that the work put into the system is equal to the internal energy of the entire system. It should be emphasized that the total energy of the system was determined (including the thermal energy that is dissipated and the energy of the friction forces of the sample material with the matrix contact surface).

Energy consumed during the extrusion process was calculated from the force F(η) vs. displacement η data. The work W(η) was calculated according to:

W(η) = F(η)∙η,(1)

and the energy used for the whole process was determined as:

(2) $$E=\int_{0}^{\eta_{e}}F(\eta )d\eta ,$$

where $\eta_{e}$—denotes final displacement.

Computations were performed through the numerical integration procedure described below.

An odd number of data was numerically integrated using the Simpson method, taking into account the unequal distance between nodes.

The Newton–Cotes formula written for three nodes of the Lagrange interpolation polynomial [48] is as follows:

(3) $$\int_{x_{1}}^{x_{3}}f\left(x\right)dx\approx H_{1}y_{1}+H_{2}y_{2}+H_{3}y_{3},$$

where $f\left(x\right)=F(x)$, F(x)—the force as a function of displacement, $y_{1}\equiv F_{1},y_{2}\equiv F_{2},y_{3}\equiv F_{3}$, N, x—displacement, mm.

The analytical computation of quadrature coefficients employing Lagrange interpolation polynomials is described here in equations. To precisely approximate integrals, one generates these coefficients by integrating basis polynomials over three locations. Precise and effective integration results depend on the formulas, which are fundamental for numerical methods such as finite element analysis or solving differential equations.

The integrals from which the quadrature coefficients are calculated analytically [49]:

(4) $$\begin{array}{l} H_{1}=\int_{x_{1}}^{x_{3}}l_{1}\left(x\right)dx=\int_{x_{1}}^{x_{3}}\frac{\left(x-x_{2}\right)\left(x-x_{3}\right)}{\left(x_{1}-x_{2}\right)\left(x_{1}-x_{3}\right)}dx=\int_{x_{1}-x_{3}}^{0}\frac{z\left(z+x_{3}-x_{2}\right)}{\left(x_{1}-x_{2}\right)\left(x_{1}-x_{3}\right)}dz\\ =\frac{z^{3}/3+\left(x_{3}-x_{2}\right)z^{2}/2}{\left(x_{1}-x_{2}\right)\left(x_{1}-x_{3}\right)}\left|\begin{array}{l} 0\\ x_{1}-x_{3} \end{array}\right.=\frac{{\left(x_{3}-x_{1}\right)}^{3}/3-\left(x_{3}-x_{2}\right){\left(x_{3}-x_{1}\right)}^{2}/2}{\left(x_{2}-x_{1}\right)\left(x_{3}-x_{1}\right)}=\\ \;\;\;\;\;\;\;\;\;\;\;\;\;\;\;\;\;\;\;\;\;\;\;\;\;\;=\frac{x_{3}-x_{1}}{x_{2}-x_{1}}\left(\frac{x_{3}-x_{1}}{3}-\frac{x_{3}-x_{2}}{2}\right), \end{array},$$

(5) $$\begin{array}{l} H_{2}=\int_{x_{1}}^{x_{3}}l_{2}\left(x\right)dx=\int_{x_{1}}^{x_{3}}\frac{\left(x-x_{1}\right)\left(x-x_{3}\right)}{\left(x_{2}-x_{1}\right)\left(x_{2}-x_{3}\right)}dx=\int_{0}^{x_{3}-x_{1}}\frac{z\left(z+x_{1}-x_{3}\right)}{\left(x_{2}-x_{1}\right)\left(x_{2}-x_{3}\right)}dz\\ =\frac{z^{3}/3+\left(x_{1}-x_{3}\right)z^{2}/2}{\left(x_{2}-x_{1}\right)\left(x_{2}-x_{3}\right)}\left|\begin{array}{l} x_{3}-x_{1}\\ 0 \end{array}\right.=\frac{{\left(x_{3}-x_{1}\right)}^{3}/3-{\left(x_{3}-x_{1}\right)}^{3}/2}{\left(x_{2}-x_{1}\right)\left(x_{2}-x_{3}\right)}=\\ \;\;\;\;\;\;\;\;\;\;\;\;\;\;\;\;\;\;\;\;\;\;\;\;\;\;=\frac{{\left(x_{3}-x_{1}\right)}^{3}}{6\left(x_{2}-x_{1}\right)\left(x_{3}-x_{2}\right)}\;, \end{array},$$

(6) $$\begin{array}{l} H_{3}=\int_{x_{1}}^{x_{3}}l_{3}\left(x\right)dx=\int_{x_{1}}^{x_{3}}\frac{\left(x-x_{1}\right)\left(x-x_{2}\right)}{\left(x_{3}-x_{1}\right)\left(x_{3}-x_{2}\right)}dx=\int_{0}^{x_{3}-x_{1}}\frac{z\left(z+x_{1}-x_{2}\right)}{\left(x_{3}-x_{1}\right)\left(x_{3}-x_{2}\right)}dz\\ =\frac{z^{3}/3+\left(x_{1}-x_{2}\right)z^{2}/2}{\left(x_{3}-x_{1}\right)\left(x_{3}-x_{2}\right)}\left|\begin{array}{l} x_{3}-x_{1}\\ 0 \end{array}\right.=\frac{{\left(x_{3}-x_{1}\right)}^{3}/3-\left(x_{2}-x_{1}\right){\left(x_{3}-x_{1}\right)}^{2}/2}{\left(x_{3}-x_{1}\right)\left(x_{3}-x_{2}\right)}=\\ \;\;\;\;\;\;\;\;\;\;\;\;\;\;\;\;\;\;\;\;\;\;\;\;\;\;=\frac{x_{3}-x_{1}}{x_{3}-x_{2}}\left(\frac{x_{3}-x_{1}}{3}-\frac{x_{2}-x_{1}}{2}\right)\;. \end{array},$$

It should be emphasized that, with an even number of experimental data, the trapezoid method was used for the last two points. The Newton–Cotes quadrature used is based on the first-degree Lagrange polynomial (a straight line passing through two points):

(7) $$\int_{x_{1}}^{x_{2}}f\left(x\right)dx\approx H_{1}y_{1}+H_{2}y_{2},$$

The integrals on the basis of which we obtain the quadrature coefficients H1 and H2 are calculated analytically:

(8) $$H_{1}=\int_{x_{1}}^{x_{2}}l_{1}\left(x\right)dx=\int_{x_{1}}^{x_{2}}\frac{x-x_{2}}{x_{1}-x_{2}}dx=\frac{{\left(x-x_{2}\right)}^{2}}{2\left(x_{1}-x_{2}\right)}\left|\begin{array}{l} x_{2}\\ x_{1} \end{array}\right.=\frac{x_{2}-x_{1}}{2},$$

(9) $$H_{2}=\int_{x_{1}}^{x_{2}}l_{2}\left(x\right)dx=\int_{x_{1}}^{x_{2}}\frac{x-x_{1}}{x_{2}-x_{1}}dx=\frac{{\left(x-x_{1}\right)}^{2}}{2\left(x_{2}-x_{1}\right)}\left|\begin{array}{l} x_{2}\\ x_{1} \end{array}\right.=\frac{x_{2}-x_{1}}{2},$$

After substituting the obtained relationships into quadrature, we obtain a formula known as the simple trapezoid formula:

(10) $$\int_{x_{1}}^{x_{2}}f\left(x\right)dx\approx \frac{1}{2}\left(x_{2}-x_{1}\right)\left(y_{1}+y_{2}\right),$$

For practical purposes, the integration interval is divided into a number of subintervals and the final integration formula assumes form [50]:

(11) $$\int_{a}^{b}f\left(x\right)dx=\frac{h}{2}\sum_{i=1}^{N}\left[f\left(x_{i}\right)+f\left(x_{i+1}\right)\right],$$

In addition, by utilizing the experimental data, a mathematical model was constructed to determine the impact of the parameter λ on the maximum force that occurs during the process of extruding a sample through a die. A comprehensive statistical analysis was conducted using the data collected from the experiments to validate and evaluate the accuracy of this model. The purpose of this investigation was to ascertain whether there is a correlation between the maximum force and the parameter λ. The study examined the importance of certain factors that affect the maximum force, how well the model matched the experimental data, and the errors in the model. It was feasible to modify die geometry and process parameters as a result of the findings of this study, which led to the possibility of beneficial applications in industry. The findings of this study helped to elucidate the processes that control the extrusion process.

## 3. Results

Based on the experimental data, the energy required to press a material sample through a die of the shape shown in Figure 2 was numerically calculated. The calculation results are presented in Table 1. As shown in Table 1, we observe the highest maximum force, as well as the highest energy needed to change the form of the sample forced through a straight hole with a diameter of 5 mm. The smallest energy changed the form of the sample pressed through a hole with a diameter of 15 mm and an angle of 60°. Then, using artificial neural networks, models were created to determine the energy used during the extrusion of the material sample. It should be emphasized that this is an innovative approach that allows for learning a nonlinear model based on experimental data, enabling the prediction of the tested value. The dataset was normalized to the [0, 1] range using min–max scaling prior to training. The input vector included two parameters: the elongation coefficient (λ) and die geometry encoded as a categorical variable. The target output was the experimentally measured energy consumption in joules. The models were trained using the Levenberg–Marquardt backpropagation algorithm with a learning rate of 0.01, a batch size of 8, and early stopping after 10 validation iterations without improvement. The MLP network input included two variables: the elongation coefficient (λ) and die geometry (numerically encoded). The output variable was the predicted energy consumption [J]. Prior to training, all data was normalized to the [0, 1] interval using min–max scaling. Training was conducted using the Levenberg–Marquardt backpropagation algorithm with a learning rate of 0.01 and early stopping after 10 validation iterations. Model variants (e.g., 2-3-1, 2-5-1, 2-9-1) were evaluated based on R^2^ correlation metrics and prediction error.

Table 2 shows the correlation coefficients for different MLP (Multi-Layer Perceptron) models used to predict the energy [J] during squeezing through a 5 mm flat matrix. The train column displays correlation values for individual network models on the training set, while the test and validation columns show correlation values on the test data set and the validation set. High values in the train and test columns indicate strong relationships, while values close to 1 indicate good prediction of energy. The validation set has high values, indicating good generalization. The MLP 2-9-1 model achieved the highest validation correlation (0.999477), with training and testing correlations of 0.974652 and 0.989879, respectively, as shown in Table 2. All models show R > 0.975 for training and R > 0.989 for testing, indicating strong predictive accuracy and generalization ability. From the above analysis of Table 2, it can be concluded that all the models are well trained (R > 0.975 for Train). The model performs equally well on the test set (R > 0.989), which indicates high generalization in the test data. As mentioned above, the best model is 7.MLP 2-9-1 as it has the highest correlation coefficient on the validation set (0.999).

**Figure 3:**
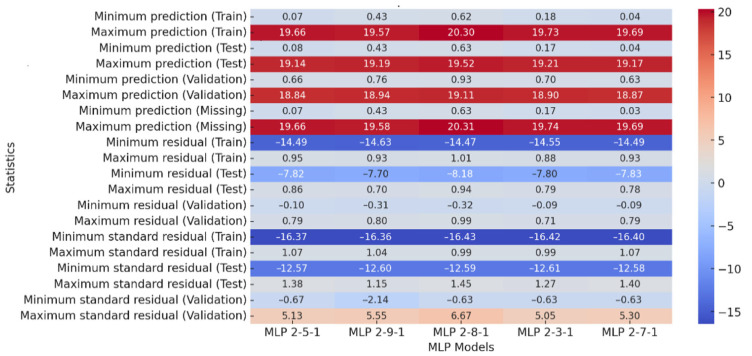
Heatmap of correlation coefficients (flat matrix for the diameter 5 mm).

Figure 3 shows prediction statistics of Multi-Layer Perceptron (MLP) models with different architectures. The training set shows the minimum predicted value for the MLP 2-5-1 model (0.07), as illustrated in Figure 3. The minimum prediction value for the 6. MLP 2-5-1 model is the lowest in the test set (0.0754), and the highest is the 9. MLP 2-3-1 model (0.7070). The maximum prediction values are similar in all models (~19.6–20.2), indicating that each model has learned to reproduce the upper range of values well.

**Figure 4:**
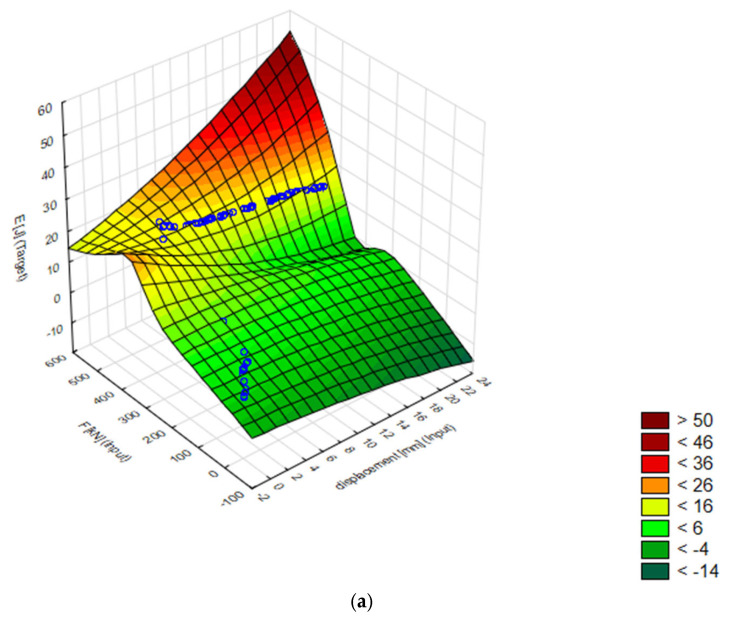

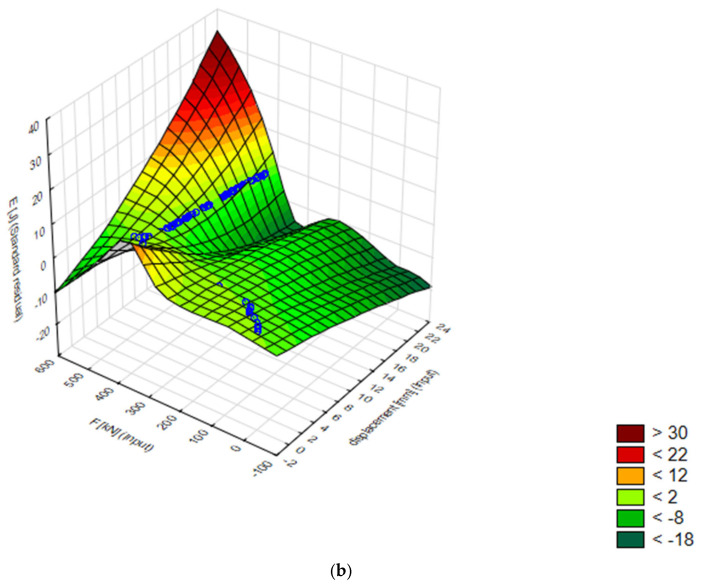
Displacement, force, and energy visualization: Target vs. standard residual analysis: (**a**) 3D energy plane in test data, (**b**) E [J] standard residual as a function of displacement [mm] and force [kN].

The 3D surfaces of energy distribution as a function of displacement and force for the best 7. MLP 2-9-1 model are presented Figure 4. Then, for comparison, a model was created to predict the energy [J] of the matrix at which the energy required to extrude the sample in the experimental data was the lowest. It is conical with an angle of α = 60° and 15 mm diameter.

Table 3 presents correlation coefficients for various Multi-Layer Perceptron (MLP) models used to predict energy during squeezing through a conical matrix. The highest correlation coefficients for train (0.961987–0.962086) indicate that the models learn dependencies well in the training set, while higher values are observed for test (0.971576–0.971920) and validation (0.977830–0.978089). The 23.MLP 2-10-1 model has the highest validation correlation (0.978089), indicating the best agreement with real values.

**Figure 5:**
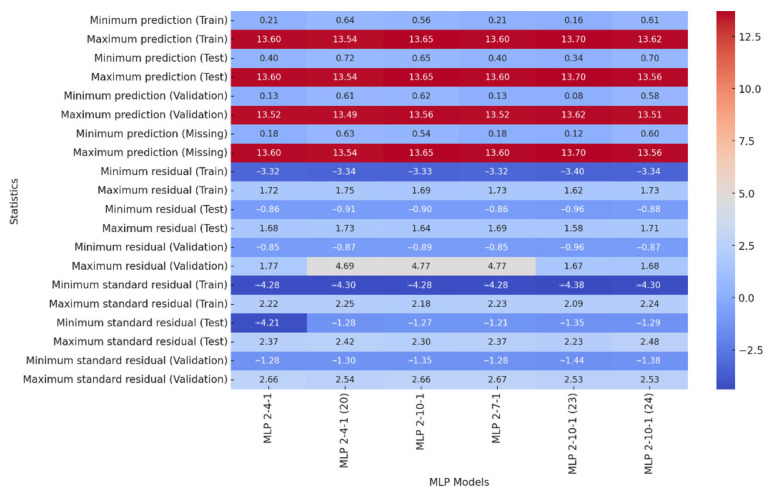
Heatmap of correlation coefficients (conical with an angle of α = 600 and 15 mm diameter).

**Figure 6:**
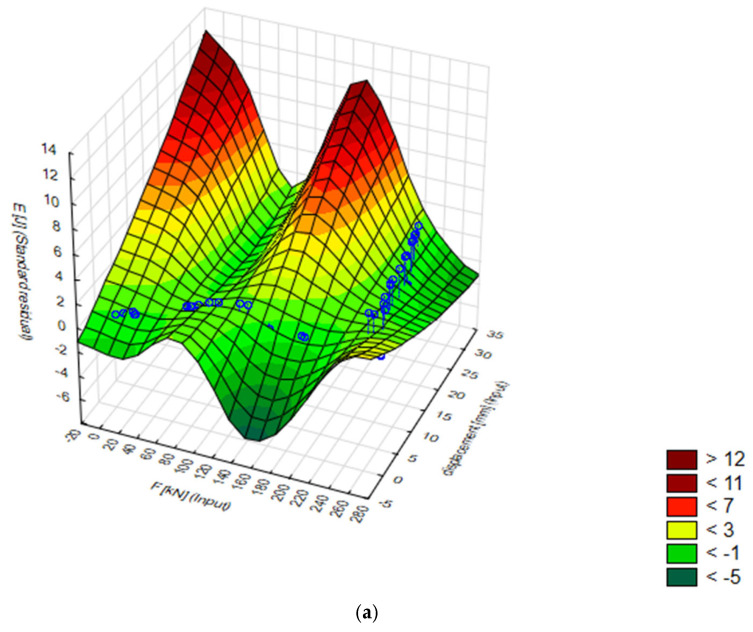

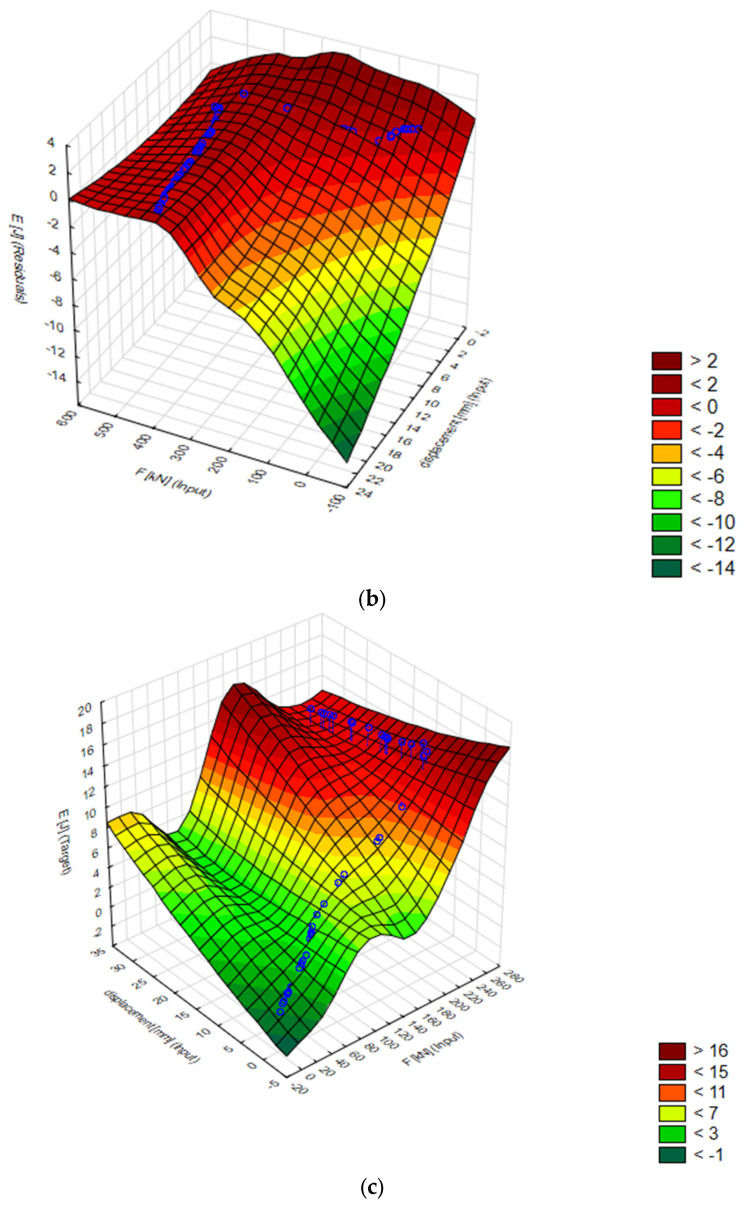
Displacement, force, and energy visualization: Target vs. standard residual analysis: (**a**) 3D energy plane in test data, (**b**) E [J] standard residual as a function of displacement [mm] and force [kN].

All models are well trained, with the best model being 23.MLP 2-10-1 due to its highest correlation coefficient on the validation set (0.978). Model prediction quality is affected by prediction range, residuals, and standardized residuals. Figure 5 shows a narrower range of predictions, suggesting less data variability or a stronger fit of the models to a specific case. The new models in Figure 6 have smaller errors, suggesting better fit to the training data and potentially better stability. The goal was to obtain models with smaller errors and more predictable results, so the models in Figure 5 perform better. After analyzing prediction statistics, the best model is 22. MLP 2-7-1, which has the smallest residuals for the training set and stable standardized residuals. The prediction ranges for the training, test, validation, and missing data sets are consistent and stable, with the smallest spreads indicating that the model performs well on different datasets.

In summary, model 22. MLP 2-7-1 stands out with the smallest prediction errors and stable results on different datasets, making it the most reliable model in this setup. Models with more neurons can lead to overfitting, and this model achieves a good trade-off between accuracy and generalization.

**Figure 7:**
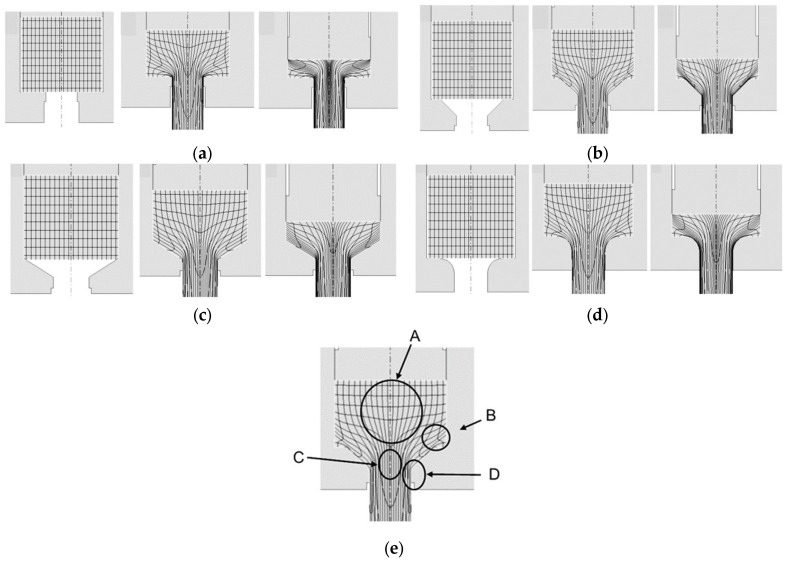
Flow line distribution and characteristic zones in forward extrusion with different die geometries: (**a**)—extrusion with flat die, (**b**)—extrusion at a conical die with angle of 60°, (**c**)—extrusion at a conical die with angle of 45°, (**d**)—extrusion with an arc die with radius R10, (**e**)—characteristic zones (from A to D) in the cross-section of the extruded profile.

Figure 7a–d show the distribution of the Lagrange grid (flow lines) in the cross-section of the forward extruded material at selected stages of computer modeling of the process, respectively using dies of the following shapes: flat, conical with an angle of 45° and 60° and arc with constant elongation coefficient λ = 6.8. For the analysis of the results, the characteristic zones of the material (from A to D) were determined according to Figure 7e.

**Figure 8:**
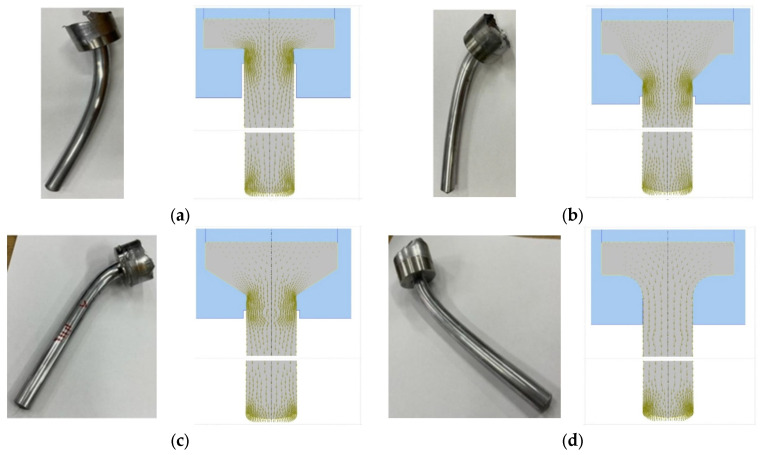
View of experimentally extruded samples and simulated material flow in forward extrusion with different die geometries (coefficient λ = 6.8): (**a**) flat; (**b**) conical with an angle of 45°; (**c**) conical with an angle of 60°; (**d**) arc with radius R10.

For the qualitative analysis of material deformation in the individual stages of the simulation, a grid system consisting of 20 vertical and 10 horizontal lines (along the OY and OX axes, respectively) was adopted. Figure 7a–d show only selected simulation steps—1, 15 and 30). No significant differences in deformation were found in the initial modeling stages. The material was initially upset until it rested against the walls of the die (zone B). In further stages of deformation, it flowed from the central part of the ingots (zone A) towards the axis of the profile (zone C). However, the most intensively distorted was the grid of flow lines near the walls of the working parts of the dies and further on the outer surface of the profiles (zone D). In the last stages of the simulation, the grid in zones C and D was deformed the most when extruded in a flat die. In the case of the remaining dies, i.e., conical and arc, the grid deformation occurred at a similar level. To confirm the correctness of the simulation results in terms of material flow kinematics and the possibility of obtaining profiles without defects under the assumed boundary conditions, an experiment was carried out with positive results. The experimentally obtained samples are shown in Figure 8. No defects in the form of possible cracks or missing fillings were found on their surface. Figure 8 shows the distribution of flow vectors in sample cross-sections using dies of various shapes with a constant coefficient λ = 6.8 in the last stage of simulation. It was found that, in the case of flat and conical dies, the highest concentration of these vectors occurs near their edges. For the arc die, the distribution of vectors is the most even in comparison to the other cases.

**Figure 9:**
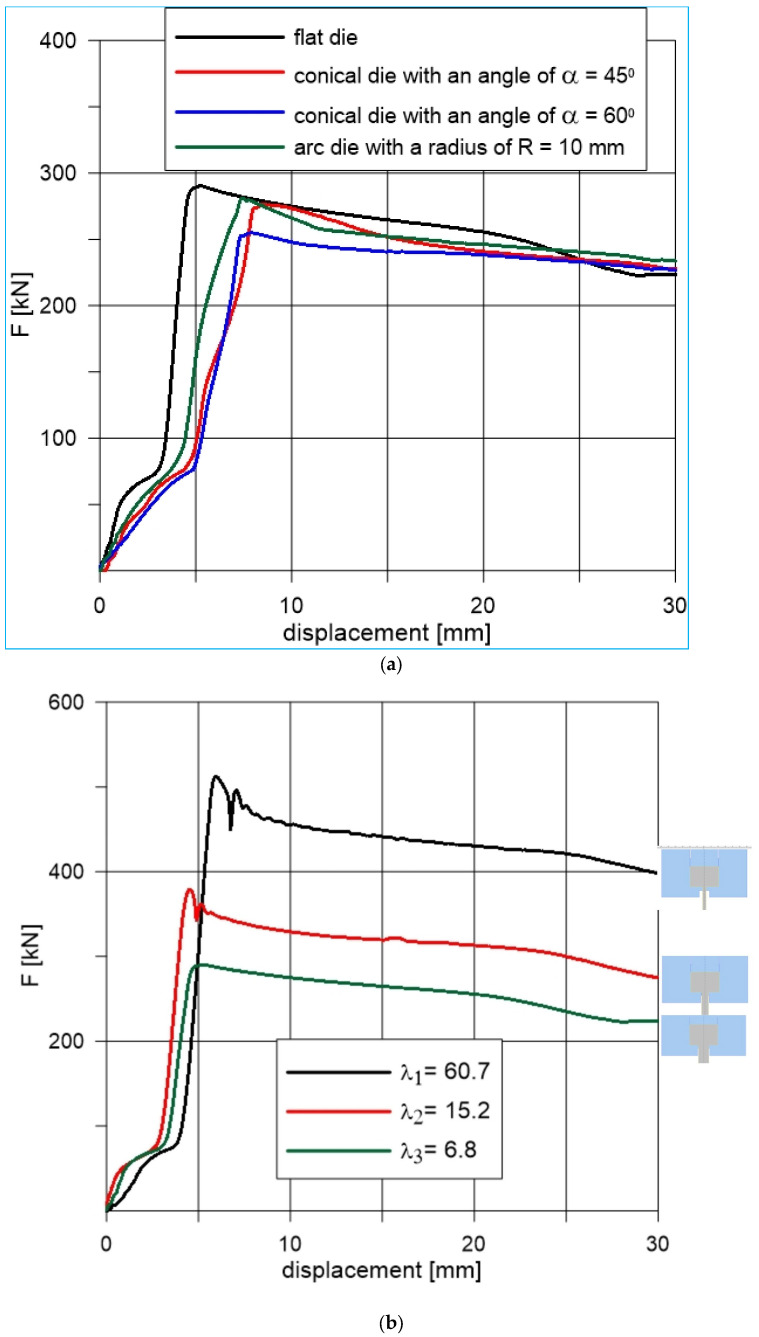
Experimental analysis of forward extrusion forces: Influence of die shape (**a**) and elongation factor (**b**).

Figure 9a shows the changes in extrusion forces obtained in the experiment as a function of punch displacement with the same elongation factor of λ = 6.8 for various shapes of dies used. The character of all force patterns, regardless of the geometry of the lower tools, was similar.

Differences occurred in the values of maximum forces and in the displacements at which these values occur. In the first stage, i.e., upsetting of material, the force increases to its maximum value. From this point on, the force decreases and the actual extrusion stage begins. The decrease occurs due to a gradual decrease in contact between the ingot and the inner wall of the container, and therefore a decrease in friction forces in this area. The AM-fabricated conical die with a 60° angle reduced extrusion force by 12% compared to conventional dies, demonstrating the effectiveness of optimized die geometry. The remaining maximum values of extrusion forces obtained for the spherical die—281 kN (at 7.44 mm) and the conical die with an angle of 45°—276.7 kN (at 8.54 mm) did not differ much from the maximum force for a flat die. These differences were approximately 3% and 5%, respectively. The maximum values of the forward extrusion forces of lead profiles occurred at various punch displacements. The smallest displacement (at which the maximum force was recorded) was 5.33 mm for a flat die, while the largest was 8.54 mm for a conical die with an angle of 45°. The remaining displacements for the arc and conical dies with an angle of 60° were similar to those for the conical die with an angle of 45° and were 7.44 mm and 7.93 mm, respectively.

The displacement values at which the maximum extrusion forces occurred were largely a consequence of the adopted depths of the input parts of the dies (the smallest depth for a flat die, about 5 mm, then 6.5 mm for an arc die and 8 mm for conical dies). In addition, an analysis of force changes was performed for different degrees of deformation. The degree of deformation was determined by the elongation coefficient λ. The individual coefficients λ = 6.8, λ = 15.2 and λ = 60.7 were obtained by extruding the material in a flat die with holes of 15 mm, 10 mm and 5 mm, respectively. Figure 9b shows the changes in extrusion forces obtained in the experiment as a function of punch displacement with the same flat shape of dies and different elongation factors. With the increase of the elongation coefficient, the maximum values of the forces increased. The greatest force value was recorded for the coefficient λ = 60.7 and amounted to 512.4 kN. It was approximately 77% higher than the maximum force value for the coefficient λ = 6.8.

Then, we performed the numerical integration of the experimental data presented in Figure 7 and Figure 8. Numerical integration of an odd number of data was performed using the formulas from Equation (8) to Equation (11), while the last two elements of an even number of data were calculated using the formula in Equation (10). Then, using the formula from Equation (11), we found the energy added to the system when the sample was pushed through a specific die shape.

Values of energy are summarized in Table 1. Values of energy were determined according to Equations (2) and (3) and are summarized in Table 1. The value of energy was determined experimentally during pressing through appropriate dies of forward extrusion. The unit of energy is:

(12) $$kN\cdot mm=1000N\cdot \frac{m}{1000}=N\cdot m=J,$$

It should be emphasized that lead is characterized by high plasticity even at room temperature, hence the plastic processing of lead at temperatures of 20–25 °C is referred to as hot plastic processing. Therefore, the energy associated with elastic changes was not determined because they are negligible in the tested range of loads and strain rates.

**Figure 10:**
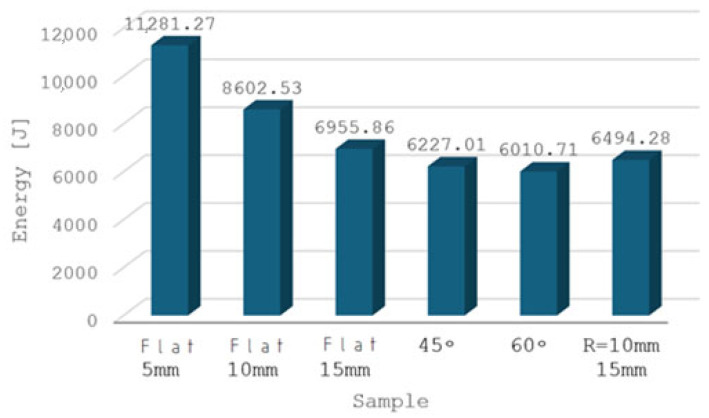
The value of energy determined experimentally during pressing through appropriate dies of forward extrusion.

Figure 10 shows the highest maximum force, as well as the highest energy needed to change the form of the sample forced through a straight hole with a diameter of 5 mm. The smallest energy changed the form of the sample pressed through a hole with a diameter of 15 mm and an angle of 60°.

This confirms the results of a previously performed computer simulation of the extrusion process using a punch with a diameter of 5 mm. In Figure 8, we see a significant density of the grid lines modeling the strong transformations taking place inside the material during the extrusion process, hence the energy needed to change the form (shape) is associated with a strong reconstruction of the internal structure.

## 4. Discussion

This study presents an in-depth approach to enhancing the extrusion process through the application of die geometry, FEM models, and artificial neural networks, aimed at evaluating energy consumption and production efficiency. The findings suggest that the efficacy of the process can be significantly improved while utilizing reduced energy by focusing on the choice of die configuration and employing sophisticated modeling methodologies.

**Figure 11:**
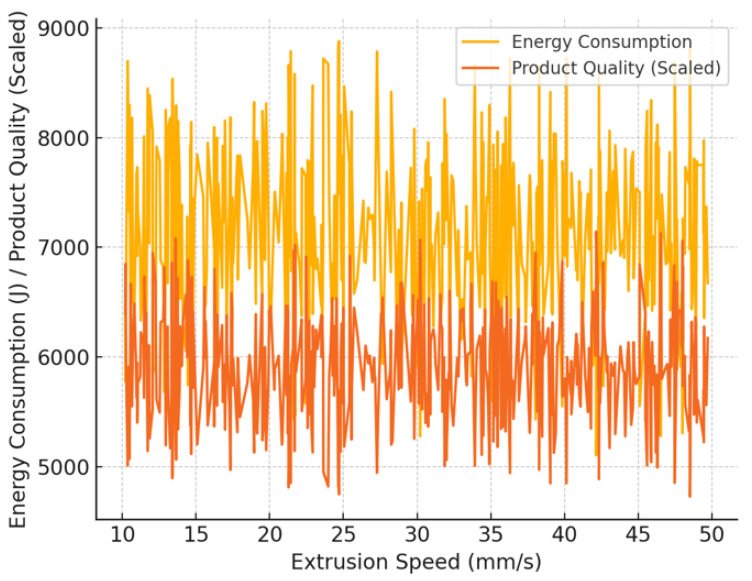
Energy consumption vs. extrusion speed.

Figure 11 illustrates how the speed of the extrusion process influences both the amount of energy consumed and the quality of the product achieved. Despite the fact that there are substantial oscillations, which indicate that the process is unstable, the energy consumption (yellow curve) increases as the extrusion speed increases. The product quality (orange curve) is more consistent, but it also depends on speed. The optimal extrusion speed range is 30–40 mm/s, which reduces energy fluctuations while maintaining high product quality. This demonstrates that, in order to obtain the highest production efficiency, a compromise must be struck between energy usage and extrusion performance. Furthermore, machine learning models based on this data achieved high prediction accuracy (R^2^ = 0.95 for the energy consumption model, R^2^ = 0.95 for the product quality model), which allows the prediction of optimal production conditions).

The results clearly show that tool geometry significantly influences both the material flow and the mechanical load distribution. This confirms the importance of geometrical optimization, not only for energy savings, but also for reducing extrusion force and stress concentration in critical zones of the die.

The numerical and experimental findings are consistent and support the validity of the proposed process modeling approach. These observations reinforce the practical value of predictive modeling in industrial applications.

**Figure 12:**
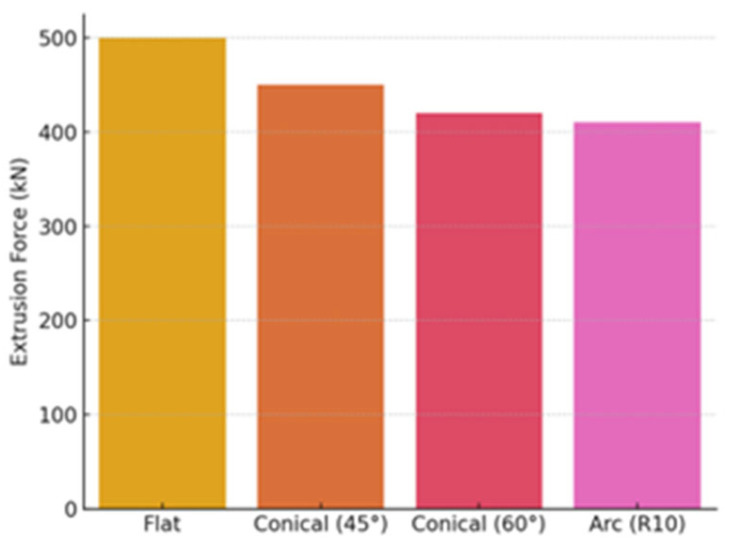
Extrusion force for different die geometries.

Figure 12 compares different die geometries in terms of their effect on extrusion force. The flat die requires the highest force (~500 kN), while the conical dies (45° and 60°) allow the force to be reduced to 450 kN and 420 kN. Even better results are achieved by the arc R10 die, which reduces the force to 410 kN, i.e., about 18% less than the flat die. These results confirm that the optimized die geometry allows significantly reduced mechanical loads and energy consumption.

**Figure 13:**
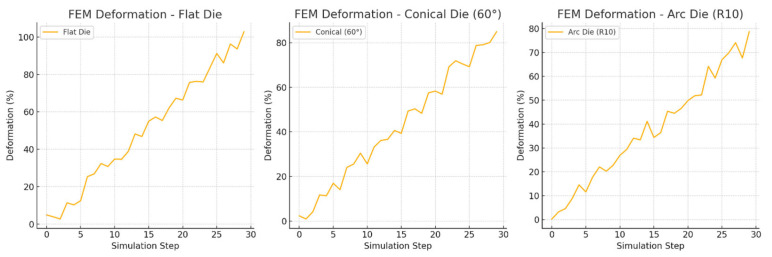
FEM deformation—flat die/conical die/arc die.

Figure 13 shows the changes in the die deformation modeled by FEM during the simulation steps. Comparing the different die geometries, it is observed that the flat die causes the highest deformation (~100%), the conical 60° die reduces the deformation to ~80%, and the arc R10 die has the lowest deformation (~75%). This shows that the optimized die shape allows for better load distribution and reduced internal stresses during the extrusion process.

The optimization methodology showed that contemporary numerical methods may be effectively applied in manufacturing processes, producing very significant outcomes in terms of mechanical load reduction, energy savings, and environmental sustainability.

By use of evolutionary algorithms to maximize die design, the most optimal parameters were found, thereby enabling the identification of the extrusion force and energy consumption decrease. The optimized die angle (88.6°), reduced surface roughness (0.12 µm), and improved cooling channel geometry not only reduced the extrusion force by about 24%, but also allowed the total energy consumption to be reduced by about 33% compared to traditional dies. These results demonstrate that numerical optimization can ensure a significant improvement in process efficiency.

## 5. Conclusions

1. The research demonstrates that the utilization of conical and arc-shaped dies can lead to a reduction in energy consumption by as much as 15% when compared to traditional flat dies. Refined die geometries are crucial for augmenting manufacturing efficacy by minimizing deformation forces and enhancing material flow. Experimental data was utilized by FEM models to produce accurate information concerning energy losses, deformation, and stress distribution. This illustrates that FEM serves as a dependable instrument for the examination and enhancement of production methodologies.
2. Furthermore, the effectiveness of artificial neural networks was evidenced; the constructed models proficiently forecasted energy consumption and extrusion forces. The MLP 2-9-1 model turned out to be the most accurate; its correlation coefficient with real data exceeded 0.999, which confirms the suitability of the model for production predictions.
3. Extrusion force analysis showed that, in the case of a flat die, the highest force is required (~500 kN), while optimized conical dies (at an angle of 45° and 60°) reduce the required force to 450 kN and 420 kN. The arc R10 die was even more effective, reducing the extrusion force to 410 kN—which is about 18% less than when using a flat die. This reduction not only saves energy but also reduces wear on tools, extending their service life.
4. In addition, extrusion speed analysis revealed that the optimal speed is 30–40 mm/s. This range leads to reduced fluctuations in energy consumption and maintains high product quality. Although higher speeds can shorten production times, they can also increase energy consumption and process instability, so it is necessary to find an optimal balance.
5. Compared to conventional dies, evolutionary algorithms applied to maximize die design allowed extrusion force to be lowered by 24% and total energy consumption to be reduced by 33%. This was accomplished by changing the die angle—ideally set at 88.6°, reducing surface roughness—down to 0.12 µm, and optimizing the shape of the cooling channels.
6. In future studies, more attention will be given to the detailed analysis of stress distribution and material behavior in the deformation zones using FEM-based stress plots and experimental microstructural validation. Additionally, the development of hybrid or multi-material extrusion dies using additive manufacturing techniques presents a promising direction for further improving energy efficiency and tool durability. The presented methodology can be directly applied to optimize industrial extrusion lines where reducing energy costs and extending tool life are critical. These results provide a strong basis for real-world implementation in automotive, aerospace, and precision metal forming industries.
7. This study provides a scientifically validated framework that combines experimental and numerical methods with machine learning to optimize energy use in metal forming. The proposed methodology can be adapted for other extrusion scenarios and extended to industrial applications, making it a meaningful contribution to the field of process optimization and sustainable engineering.

## Figures and Tables

**Table 1:** Summary of energy values at different die shapes and elongation factors.

Shape of Die	Elongation Factor λ	Energy, J
Flat with 15 mm diameter	6.8	6955.86
Flat with 10 mm diameter	15.2	8602.53
Flat with 5 mm diameter	60.7	11,281.27
Conical with an angle of α = 45° and 15 mm diameter	6.8	6227.01
Conical with an angle of α = 60° and 15 mm diameter	6.8	6010.71
Arc with a radius of *R* = 10 mm and 15 mm diameter	6.8	6494.28

**Table 2:** Correlation coefficients (flat matrix for the diameter 5 mm).

Model	E [J] Train	E [J] Test	E [J] Validation
MLP 2-5-1	0.975134	0.989473	0.999375
MLP 2-9-1	0.974652	0.989879	0.999477
MLP 2-8-1	0.975293	0.988584	0.999348
MLP 2-3-1	0.975131	0.9896	0.999476
MLP 2-7-1	0.975183	0.989485	0.999429

**Table 3:** Correlation coefficients (flat matrix for the diameter 5 mm).

Model	E [J] Train	E [J] Test	E [J] Validation
MLP 2-4-1	0.962086	0.971892	0.977869
MLP 2-4-1	0.961987	0.971576	0.97783
MLP 2-10-1	0.961999	0.971605	0.977839
MLP 2-7-1	0.962086	0.971888	0.977857
MLP 2-10-1	0.962017	0.97192	0.978089
MLP 2-10-1	0.962016	0.971628	0.977909

## Data Availability

No new data was created.
